# Field-specific nutrient management using Rice Crop Manager decision support tool in Odisha, India

**DOI:** 10.1016/j.fcr.2019.107578

**Published:** 2019-09-01

**Authors:** Sheetal Sharma, K.K. Rout, C.M. Khanda, Rahul Tripathi, Mohammad Shahid, Amarash Nayak, Swetapadma Satpathy, Narayan Chandra Banik, Wasim Iftikar, Nabakishore Parida, Vivek Kumar, Amit Mishra, Rowena L. Castillo, Theresa Velasco, Roland J. Buresh

**Affiliations:** a International Rice Research Institute, India; b International Rice Research Institute, PO Box 7777, Metro Manila, Philippines; c CIMMYT, India; d Odisha University of Agriculture and Technology (OUAT), Bhubaneswar, India; e National Rice Research Institute (NRRI), Cuttack, Odisha, India

**Keywords:** ACZ, agro-climatic zones, ANB, added net benefit, BFR, blanket fertilizer recommendation, DAT, days after transplanting, FFP, farmers’ fertilizer practice, FN, fertilizer N rate, GRF, gross return above fertilizer cost, GY, grain yield, GY_R_, historical grain yield reported by farmer, GY_T_, target grain yield, NMR, Nutrient Manager for Rice, NOPT, nutrient omission plot technique, PFP, partial factor productivity of added N, RCM, Rice Crop Manager, SSNM, site-specific nutrient management, TFC, total fertilizer cost, Fertilizer recommendation, On-farm research, Rice, Rice crop manager, Site-specific nutrient management

## Abstract

The requirement of rice (*Oryza sativa* L.) for fertilizer can depend on crop and soil management practices, which can vary among fields within a rice-growing area. A web-based decision support tool named Rice Crop Manager (RCM) was developed previously to calculate field-specific rates of fertilizer N, P, and K for rice in Odisha State in eastern India. We compared field-specific nutrient management calculated by RCM with farmers’ fertilizer practice (FFP) and a blanket fertilizer recommendation (BFR), which used a uniform 80 kg N ha^–1^, 17 kg P ha^–1^, and 33 kg K ha^–1^. A total of 209 field trials were conducted in two seasons (kharif and rabi) for two years across ten districts in six agro-climatic zones. Grain yield was consistently higher with fertilization recommended by RCM than with FFP. Higher yield with RCM was attributed to a combination of applying more of the total fertilizer N at the critical growth stage of panicle initiation, applying more fertilizer N in kharif, and applying zinc. The RCM recommendation frequently increased yield compared to BFR as a result of improved N management, which included the adjustment of N rate for a target yield set slightly higher than historical yield reported by a farmer. Fertilization based on RCM rather than BFR reduced the risk of financial loss. The effectiveness of an RCM recommendation relative to BFR and FFP was consistent across rice varieties with different growth duration, irrigated and rainfed rice, and three categories of soil clay content. The RCM recommendation failed to increase yield relative to BFR in one of the six agro-climatic zones, where a higher rate of fertilizer P and/or K was apparently required. The nutrient management calculations used by RCM can be improved as new information and research findings become available. Experiences with RCM in Odisha can help guide the development of comparable nutrient management decision tools in other rice-growing areas.

## Highlights

• Rice Crop Manager (RCM) calculated field-specific nutrient management in Odisha.
• Rice yield was greater with an RCM recommendation than farmers' fertilizer practice (FFP).
• RCM had less risk of financial loss than a blanket fertilizer recommendation (BFR).
• RCM was effective across rice varieties, irrigated and rainfed rice, and soils.
• Use of RCM recommendation rather than BFR improved the rate of fertilizer N.

## 1 Introduction

India is the second largest rice-producing country after China (GRiSP, 2013), and much of the rice within India is produced and consumed in eastern India (Singh and Singh, 2000). Eastern India has, however, lagged behind the rest of India in rice yields and prosperity for farmers (Jha et al., 2012). Rice production in eastern India, as compared to the rest of India, can often be more constrained by drought, floods, salinity, low soil fertility, and insufficient or inefficient use of fertilizers (Singh and Singh, 2000). More effective use of fertilizer is recognized as an opportunity for increasing rice production in eastern India.

Fertilizer recommendations for rice in Asia, including India, have historically often entailed a uniform, blanket application of nutrients across an area or domain of rice production. Site-specific nutrient management (SSNM) for rice was developed starting in the 1990s to enable adjustment in fertilizer management for spatial and temporal variability in the need of a crop for supplemental application of N, P, and K (Dobermann et al., 2002, 2004b). SSNM used principles from the model QUEFTS (Janssen et al., 1990) to provide algorithms for calculating fertilizer requirements adjusted for specific target yields and rice-growing practices (Witt and Dobermann, 2004; Buresh et al., 2010). The International Rice Research Institute (IRRI) in collaboration with national partners across Asia incorporated these SSNM-based algorithms into a web-based decision support tool, named Nutrient Manager for Rice (NMR), which calculated rates and times of fertilizer application for individual rice fields (Buresh et al., 2014).

Field-specific fertilizer recommendations calculated by NMR increased yield and net income compared to the existing farmers’ fertilizer practice in on-farm research with irrigated rice in southern India (Sharma et al., 2019). Sharma et al. (2019) also compared NMR to an existing recommendation for a blanket application of fertilizer in the absence of soil testing. NMR recommendations did not increase yield compared to the blanket fertilizer recommendation (BFR), but NMR reduced fertilizer cost and risk of financial loss and increased fertilizer use efficiency compared to BFR. Beginning in 2013, IRRI converted NMR into a web-based Rice Crop Manager (RCM) (Buresh et al., 2019), which provided advice on crop management practices in addition to nutrient management. The component of field-specific fertilizer management calculated by RCM has been shown in on-farm research to increase yield and net income compared to existing farmers’ fertilizer practices in West Africa (Saito et al., 2015) and the Philippines (Banayo et al., 2018b). Field-specific fertilizer recommendations calculated by RCM were not compared in these studies to an existing fertilizer recommendation such as a BFR.

Within eastern India, Odisha is one of the main rice-producing states (Dar et al., 2017), but average rice yields in Odisha are distinctly lower than the national average (Das, 2012). Much of the rice in Odisha is produced in small landholdings varying in crop management practices and constraints such as drought and floods, which influence rice yields and the need for supplemental nutrients (Singh and Singh, 2000). This spatial variability among rice fields could make Odisha well suited for field-specific nutrient management as calculated by RCM with rates and times of fertilizer application adjusted for a target yield, rice variety, and crop management of a specific field rather than a uniform blanket fertilizer recommendation.

Our objective was to verify the performance of SSNM-based field-specific nutrient management calculated by RCM relative to a uniform blanket fertilizer recommendation (BFR) and farmers’ fertilizer practice (FFP). Research was across a range of rice-growing conditions in Odisha to determine the effects of agro-climatic zone, soil, cropping season, water regime, and rice variety on the ability of a nutrient management recommendation calculated and deployed by RCM to increase rice yield and net income and to reduce risk of financial loss. The BFR used nutrient rates consistent with management practices promoted within Odisha (DAFP, 2007; 2008).

## 2 Materials and methods

### 2.1 Site characterization

**Fig. 1:**
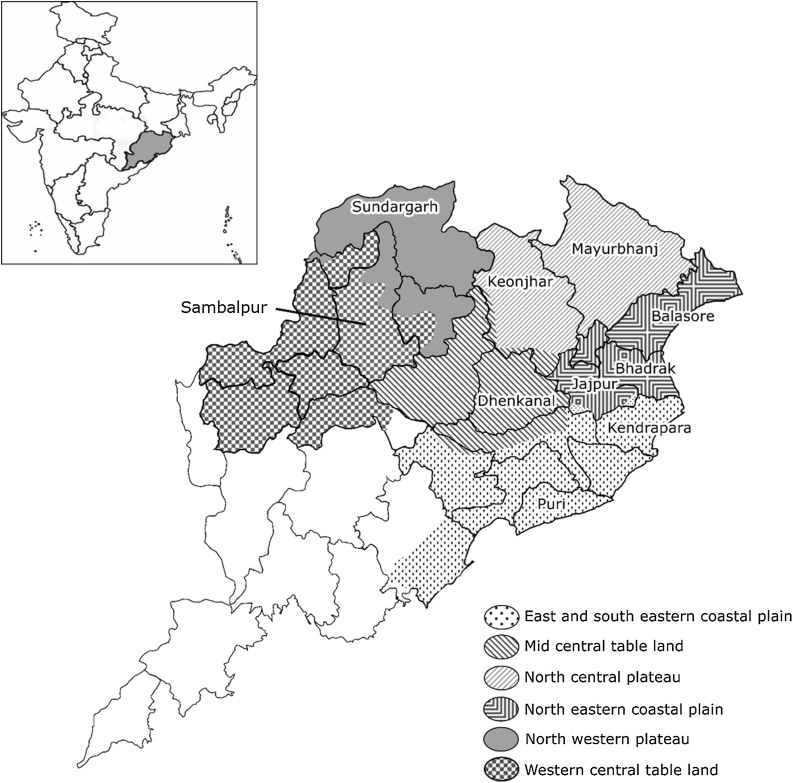
Map for Odisha State in India showing the ten districts and six agro-climatic zones with field trials.

On-farm trials were conducted with rice in two kharif (wet) cropping seasons (2014 and 2015) and two rabi (dry) cropping seasons (2013–2014 and 2014–2015). The kharif is from July–October during the southwest monsoon, and rabi is from October–March during the dry mild winter. The climate is tropical with high temperature, high humidity, and medium to high rainfall during kharif. The trials were distributed across ten major rice-growing districts in six of the ten agro-climatic zones (ACZ) in Odisha (Fig. 1, Supplement Table 1). Soils range from alluvial deltaic soils in coastal plains, mixed red and black soils in central table lands, and red and yellow soils with low fertility in the northern plateau. Trials in kharif were both irrigated and rainfed, but all trials in rabi were irrigated.

### 2.2 Experimental design

Each of 209 trials was conducted with transplanted rice in a farmer’s field divided into three plots with each plot ranging between 160–800 m^2^. The following three unreplicated treatments were randomly assigned to a plot in a trial: field-specific nutrient management calculated by RCM, BFR, and FFP. Each plot was surrounded by an earthen levee to prevent movement of fertilizer in and out of plots.

The farmer for each of the 209 field trials was interviewed before the start of the cropping season to determine a farmer’s planned time for each fertilizer application and planned amounts of fertilizer sources selected by the farmer for each application. This planned fertilizer practice was unique for each field trial and served as the FFP treatment. It was fixed before a farmer was exposed to an RCM recommendation to ensure the farmers’ fertilizer practice was not influenced by RCM.

Each farmer was then interviewed before crop establishment with RCM (http://webapps.irri.org/in/od/rcm) to collect information on the farmer’s field location, size of the field, rice variety, anticipated age of seedlings at transplanting, water management (irrigated or rainfed), rice yield in previous years with the same or similar variety, portion of above-ground residues from the previous crop retained in the field, and choice of fertilizer sources. This information was used by RCM to calculate a field-specific fertilizer recommendation to achieve a target yield set by RCM. This RCM recommendation included rates and times for application of fertilizer sources selected by the farmer. It was unique for each field trial and served as the RCM treatment. RCM typically set the target yield higher than the historical yield reported by the farmer during the RCM interview, but RCM also adjusted target yield downward when transplanting was delayed beyond a critical date, and RCM limited upward adjustment in yield for low-yielding rice varieties (Sharma et al., 2019). As a result, target yield was less than historical yield reported by the farmer in 12% of the field trials in our study. Target yield for RCM in our study was within 3.0–6.5 Mg ha^–1^.

RCM used a yield gain approach to calculate fertilizer N rate for a target yield as the estimated increase in yield from applied N divided by a target agronomic efficiency of added fertilizer N (Buresh, 2015; Buresh et al., 2019). The estimated increase in yield from applied N as a function of target yield can be calibrated using results from nutrient omission plot technique (NOPT) trials (Witt et al., 2007) as reported for NMR in Tamil Nadu, India (Sharma et al., 2019). Results from NOPT trials were not available for Odisha when our study evaluated RCM in 2013–2015. The RCM evaluated in our study consequently calculated fertilizer N rate using an increase in yield from applied N that was estimated from NOPT trials conducted outside Odisha. With this approach, the calculated fertilizer N rate with RCM increased linearly from 57 kg N ha^–1^ at target yield = 3 Mg ha^–1^ to 123 kg N ha^–1^ at target yield = 6 Mg ha^–1^. The corresponding values for NMR in Tamil Nadu reported by Sharma et al., 2019) were 55 kg N ha^–1^ at target yield = 3 Mg ha^–1^ to 139 kg N ha^–1^ at target yield = 6 Mg ha^–1^.

Fertilizer N with RCM was applied in three splits: early vegetative stage, mid-tillering, and panicle initiation. Mid-tillering and panicle initiation are critical stages for application of fertilizer N (Peng and Cassman, 1998), but the number of days after transplanting to reach these critical stages depends on growth duration of the rice variety and age of transplanted seedlings. The farmers in our study selected 24 different rice varieties varying in growth duration and prevalence between the kharif and rabi seasons (Supplement Table 2). RCM used information collected from the farmer on name of variety and age of transplanted seedlings and then accessed information on growth duration of the selected variety, which was stored in a database, to calculate a recommendation with days after transplanting for each fertilizer N application (Sharma et al., 2019).

RCM calculated fertilizer P rate using a nutrient input-output balance, in which P required for the target yield of a specific field was the difference between the estimated P taken up by the mature crop and the P added with retained residues from the previous crop (Buresh et al., 2010, 2019). In our study, the residue of the previous crop was manually cut slightly above ground level and removed by farmers. RCM calculations assumed only 15% of the total above-ground crop biomass after harvest of grain was retained in the field. RCM calculations also assumed harvested grain contained 70% of total plant P (Dobermann and Fairhurst, 2000). All fertilizer P was applied basal.

RCM used a yield gain approach to calculate fertilizer K rate as the estimated amount of additional K taken up by the crop to achieve a target yield divided by a recovery efficiency of added fertilizer K (Buresh et al., 2010), which was set to 0.44 kg kg^–1^ (Witt and Doberman, 2004). Results from NOPT trials in Odisha were not available for calibration when our study evaluated RCM in 2013–2015, but Buresh et al. (2010) reported a mean 12% yield gain from applied K across 525 NOPT trials conducted with irrigated rice in four Asian countries. The RCM evaluated in our study consequently calculated fertilizer K rate using a slightly higher 15%, rather than 12%, increase in yield from applied K in order to calculate a higher rate of fertilizer K with less risk of underapplication.

Fertilizer K was all applied basal when total K rate was ≤33 kg K ha^–1^ and it was applied 50% basal and 50% at panicle initiation when total K rate was >33 kg K ha^–1^. The time interval for the basal application of N, P, and K varied with duration of rice variety. It ranged from within 5 days after transplanting for the shortest duration varieties to within 21 days after transplanting for longest duration varieties. All P and K rates are expressed on an elemental basis.

The BFR was determined based on an existing Odisha State recommendation for high-yielding rice varieties in lowlands in kharif (DAFP, 2008) and medium duration varieties on low fertility soil in rabi (DAFP, 2007). The BFR was 80 kg N ha^–1^, 17 kg P ha^–1^, and 33 kg K ha^–1^. Fertilizer N was distributed 20% basal, 50% at tillering, and 30% at panicle initiation. All fertilizer P was applied basal, and fertilizer K was applied 50% basal and 50% at panicle initiation.

Urea was the main source of fertilizer N, muriate of potash (KCl, 0-0-60) was the main source of fertilizer K, and diammonium phosphate (DAP, 18-46-0) was the main source of fertilizer P across RCM, FFP, and BFR treatments (Supplement Table 3). Compound NP fertilizers (28-28-0 and 20-20-0) or NPK fertilizers (10-26-26) were applied in several trials with RCM and FFP (Supplement Table 3). Zinc sulfate at an average rate of 25 kg ha^–1^ was applied in the basal fertilizer application for RCM and BFR in all trials, thereby ensuring differences in crop performance and yield between RCM and BFR treatments were not due to zinc.

Zinc sulfate was applied in FFP as indicated by each farmer during an interview before the start of the cropping season. Most farmers reported no use of zinc, and the FFP treatment received zinc sulfate in only 5% of the trials. The application of each farmer’s reported rate of zinc sulfate ensured the total fertilizer cost for FFP reflected each farmer’s reported fertilizer use. However, because of the difference in zinc sulfate rate between FFP and other treatments, zinc could be a factor responsible for differences in crop performance and yield between FFP and either the RCM or BFR treatment.

Researchers managed all fertilizer applications for all treatments in order to avoid change or bias by the farmers. Except for fertilizer application, all other management practices in a trial including land preparation, variety, crop establishment, residue management, water management, and crop protection practices were determined and implemented by the farmer and were identical for the three treatments in a trial. The differences in crop performance and yield within a field trial consequently reflect only differences in fertilizer management.

### 2.3 Measurements and analyses

Composite soil samples were collected from 0 to 15 cm depth from each trial before crop establishment and fertilizer application. Soil samples were air dried and crushed. Soil pH was determined in water with a 1:2 soil–water ratio, organic C was determined by the Walkley-Black chromic acid wet oxidation method, available N was determined by distillation with alkaline potassium permanganate, Olsen P was determined by extraction with 0.5 M sodium bicarbonate at pH 8.5, exchangeable K was determined by extraction with 1 M ammonium acetate at pH 7, and Zn was determined by extraction with 0.005 M diethylenetriamine pentaacetic acid (DTPA) + 0.01 M calcium chloride + 0.1 M triethanolamine (TEA) at pH 7.3. Soil texture was estimated by the feel method (NRCS Soils (United Sates Department of Agriculture, Natural Resources Conservation Services Soils), 2019)

Grain was harvested at physiological maturity from three randomly selected 10 m^2^ areas in each plot. Grain moisture content was determined with a moisture meter, and grain yield was expressed at 14% water content.

The cost for each fertilizer source used in a field plot was determined from the amount of the fertilizer source applied and the farmgate price for the source, averaged across all districts and seasons. The average prices were 0.09 US$ kg^–1^ for urea, 0.26 US$ kg^–1^ for muriate of potash (MOP), 0.38 US$ kg^–1^ for DAP, 0.14 US$ kg^–1^ for single superphosphate, 1.08 US$ kg^–1^ for zinc sulfate, 0.38 US$ kg^–1^ for 28-28-0, 0.44 US$ kg^–1^ for 20-20-0, and 0.36 US$ kg^–1^ for 10-26-26. The total fertilizer cost (TFC) for a field plot was the sum of the costs for all applied fertilizer sources. Prices and financial analyses are reported in US$ using an exchange rate of 1 US$ = 65 Indian rupees (INR).

Gross return was calculated as the product of grain yield and farmgate price of unmilled rice averaged across districts within a season. The average farmgate price was 0.21 US$ ha^–1^ for 2013–2014 rabi, 2014 kharif, and 2014–2015 rabi seasons. It was 0.22 US$ ha^–1^ for 2015 kharif. The farmgate price of unmilled rice approximated the minimum support price fixed by the Government of India (DAC, 2015). Gross return above fertilizer cost (GRF) was calculated as the difference between gross return and the total cost of fertilizer (Dobermann et al., 2004a). Added net benefit for RCM relative to BFR and FFP was calculated as the difference in GRF between RCM and the other treatment. Partial factor productivity of added N (PFP) expressed in kg grain per kg N was calculated as follows

PFP = 1000 × GY/FN

where FN is fertilizer N expressed in kg ha^–1^ and GY is grain yield expressed in Mg ha^–1^ (Dobermann, et al., 2004a).

All data were analyzed using mixed models fitted separately for each of the response variables—rate of fertilizer (N, P, and K), yield, TFC, PFP, and added net benefit. According to the hypotheses being tested, the factors of treatment, ACZ, season, soil clay content, water regime, and duration of rice variety were included in the model as fixed effects. Interactions between factors were included as fixed effects and farmer field trial was included as a random effect. Post-hoc tests for differences between means were conducted using Tukey tests for pairwise differences between more than two means, and Dunnett's tests for pairwise differences between two means at alpha = 0.05 level of significance. Analyses were conducted in the R programming language and environment (R Core Team, 2016). Mixed models were implemented using the R package *lmer* (Bates et al., 2015), and post-hoc tests were implemented in the R package *emmeans* (Lenth, 2018).

## 3 Results

### 3.1 Site characteristics

Mean soil properties for all 209 field trials and for field trials in each of the six ACZ are shown in Table 1. Mean soil pH for the six ACZ ranged from 5.0 to 6.1 and was lowest for the ten trials in the mid central table land zone. Mean exchangeable soil K, which ranged from 0.15 to 0.34 cmol_c_ kg^–1^, was also lowest in the mid central table land zone. Organic C and available N were lowest for the eleven trials in the north western plateau zone. Mean Olsen P and extractable zinc were somewhat comparable across ACZ. The variability within ACZ for measured soil properties was relatively large as indicated by the standard deviations (Table 1).

**Table 1:** Soil characteristics for field trials conducted across six agro-climatic zones (ACZ) in Odisha, India.

Characteristic^a^	All locations	East and south eastern coastal plain	Mid central table land	North central plateau	North eastern coastal plain	North western plateau	Western central table land
Field trials (n)	209	66	10	58	54	11	10
pH	5.8 (0.7)^b^	6.1 (0.7)	5.0 (0.3)	5.7 (0.5)	5.7 (0.8)	5.5 (0.3)	6.1 (0.6)
Organic C (g kg^–1^)	7.4 (2.9)	6.4 (1.7)	5.7 (1.0)	9.5 (3.0)	6.6 (2.0)	5.4 (2.5)	9.2 (5.3)
Available N (mg kg^–1^)	84 (29)	83 (38)	85 (21)	78 (20)	91 (22)	73 (19)	104 (34)
Olsen P (mg kg^–1^)	7 (4)	7 (5)	8 (2)	6 (2)	8 (4)	5 (3)	9 (5)
Exch K (cmol_c_ kg^–1^)	0.24 (0.14)	0.21 (0.10)	0.15 (0.04)	0.27 (0.17)	0.24 (0.12)	0.34 (0.28)	0.29 (0.08)
Zn (mg kg^–1^)	1.3 (1.0)	1.2 (0.9)	1.4 (1.0)	1.6 (1.3)	1.3 (1.0)	1.0 (0.6)	1.0 (0.6)

a Reported values include trials in both kharif and rabi seasons. pH determined in 0.01 M CaCl_2_, organic C determined by Walkley-Black method, available N determined by alkaline permanganate extraction, Olsen P determined by bicarbonate extraction, exchangeable (exch) K determined by extraction with ammonium acetate, and Zn determined by DTPA extraction.

b Values in parentheses are standard deviations.

### 3.2 Evaluation across two seasons

The performance of RCM relative to FFP and BFR was first examined across two seasons (kharif and rabi) and two years for each season (Table 2). Mean fertilizer N rates with RCM recommendations were comparable across seasons and years (98–106 kg ha^–1^), and they were higher than the uniform application of 80 kg N ha^–1^ with BFR in each season and year. Fertilizer N rates averaged 33 and 25 kg ha^–1^ higher with RCM than FFP in kharif because of relatively low N use with FFP in kharif. Mean fertilizer N rates in rabi, in contrast, were comparable for RCM recommendations and FFP (Table 2); but the range in fertilizer N rates in rabi was much larger for FFP (14–252 kg ha^–1^) than RCM (54–143 kg ha^–1^) (Supplement Table 4).

**Table 2:** Rates of N, P, and K fertilizer, total fertilizer cost (TFC), measured grain yield, partial factor productivity of added N (PFP), and added net benefit (ANB) for field-specific nutrient management provided by Rice Crop Manager (RCM), farmers’ fertilizer practice (FFP), and blanket fertilizer recommendation (BFR); historical grain yield reported by farmers (GY_R_); and target grain yield with RCM (GY_T_) across two seasons (kharif and rabi) and two years in Odisha, India.

Parameter	Treatment or contrast	Kharif		Rabi	
		2014	2015	2013–2014	2014–2015
Trials (n)		84	24	39	62
N rate (kg ha^–1^)^a^	RCM	103 a^b^	106 a	102 a	98 a
	FFP	70 a	81 ab	101 c	94 bc
	RCM–FFP^c^	33^***^	25^***^	1 ns	4 ns
P rate (kg ha^–1^)^a^	RCM	14 a	12 a	11 a	14 a
	FFP	20 a	18 a	28 b	24 b
	RCM–FFP	–6^***^	–6^**^	–17^***^	–11^***^
K rate (kg ha^–1^)^a^	RCM	28 b	34 b	19 a	27 ab
	FFP	39 a	47 a	47 a	44 a
	RCM–FFP	–11^***^	–12^**^	–28^***^	–17^***^
TFC (US$ ha^–1^)	RCM	88 a	90 a	78 a	85 a
	FFP	70 a	90 bc	100 c	85 b
	BFR	90 a	87 a	96 a	90 a
	RCM–FFP	17^***^	0 ns	–22^***^	–1 ns
	RCM–BFR^d^	–3 ns	3 ns	–18^***^	–5 ns
	BFR–FFP^e^	20^***^	–3 ns	–4 ns	4 ns
Grain yield (Mg ha^–1^)	RCM	5.5 b	4.8 a	5.4 ab	4.9 a
	FFP	4.7 ab	4.2 a	5.2 b	4.4 a
	BFR	5.2 b	4.6 a	5.0 ab	4.7 a
	RCM–FFP	0.8^***^	0.6^***^	0.3^*^	0.5^***^
	RCM–BFR	0.3^***^	0.3 ns	0.4^***^	0.2^*^
	BFR–FFP	0.5^***^	0.3^*^	–0.2 ns	0.3^***^
PFP (kg kg^–1^)	RCM	54 a	46 a	54 a	51 a
	FFP	73 b	54 a	61 a	55 a
	BFR	65 a	56 a	63 a	59 a
	RCM–FFP	–19^***^	–9 ns	–7 ns	–5 ns
	RCM–BFR	–11^***^	–10 ns	–8*	–8^*^
	BFR–FFP	–8^**^	2 ns	2 ns	3 ns
ANB (US$ ha^–1^)	RCM–FFP	150 b	129 ab	76 a	110 ab
	RCM–BFR	65 a	54 a	106 a	50 a
	Diff^f^	85^***^	75^**^	–30 ns	60^***^
GY_R_ (Mg ha^–1^)^g^		4.6 b	4.4 b	3.7 a	4.4 b
	Diff from FFP yield^h^	–0.1 b	0.2 b	–1.5 a	0.0 b
GY_T_ (Mg ha^–1^)		5.1 b	4.9 b	4.2 a	4.9 b
	Diff from RCM yield^i^	–0.5 b	0.1 c	–1.2 a	0.0 c

ns = not significant (P > 0.05). ^*^, ^**^, and ^***^ indicate significance at the 0.05, 0.01, and 0.001 probability levels, respectively.

a Fertilizer applied with BFR: N = 80 kg ha^–1^, P =17 kg ha^–1^, and K =33 kg ha^–1^.

b Means within a row followed by the same lowercase letter are not different at P ≤ 0.05.

c RCM–FFP = Difference between RCM and FFP.

d RCM–BFR = Difference between RCM and BFR.

e BFR–FFP = Difference between BFR and FFP.

f Diff = Difference between RCM–FFP and RCM–BFR.

g Number for trials: 2014 = 82, 2015 = 24, 2013–2014 = 39, and 2014–2015 = 60.

h Diff from FFP yield = Difference between the historical grain yield reported by farmer (GY_R_) and the measured yield with FFP.

i Diff from RCM yield = Difference between the RCM target grain yield (GY_T_) and the measured yield with RCM.

Mean fertilizer P and K rates were consistently lower with RCM recommendations than FFP across seasons and years (Table 2). The mean reductions with RCM compared to FFP were 6–17 kg P ha^–1^ and 11–28 kg K ha^–1^. All trials received P and K with RCM; but with FFP, some trials received no P or K and some trials received very high rates of P or K. Fertilizer P rates across the two seasons ranged from 0 to 64 kg ha^–1^ with FFP as compared to 6–21 kg ha^–1^ with RCM (Supplement Table 4). Fertilizer K rates across the two seasons were 0–156 kg ha^–1^ with FFP as compared to 12–49 kg ha^–1^ with RCM.

Total fertilizer cost (TFC) was unaffected by season and year for RCM (78–90 US$ ha^–1^) and BFR (Table 2). The TFC was comparable for RCM and BFR, except for rabi 2013–2014 when TFC was 18 US$ ha^–1^ lower for RCM largely because of less fertilizer K use with RCM in that season. Mean TFC varied across seasons and years with FFP (70–100 US$ ha^–1^), and difference between RCM and FFP consequently varied across seasons and years. The TFC with RCM was 17 US$ ha^–1^ higher than for FFP in kharif 2014, 22 US$ ha^–1^ lower than for FFP in rabi 2013–2014, and comparable to FFP in other seasons and years.

Zinc sulfate followed by diammonium phosphate (DAP) accounted for the highest portions of the total fertilizer cost with RCM (Table 3). Zinc sulfate, which was applied as a uniform rate in RCM and BFR, represented 32% of the total fertilizer cost with RCM. Diammonium phosphate (DAP) and muriate of potash (MOP) accounted for 30% and 16%, respectively, of the total fertilizer cost. Urea was the source applied in the largest quantity with RCM, but it represented only 22% of the fertilizer cost because of the subsidized low price per kg for urea compared to DAP, MOP, and zinc sulfate.

**Table 3:** Costs for urea, muriate of potash (KCl, 0-0-60), diammonium phosphate (DAP), and zinc sulfate (expressed as percentage of total fertilizer cost) for field-specific nutrient management provided by Rice Crop Manager (RCM) in 178 field trials across two seasons in Odisha, India.

Fertilizer^a^	Fertilizer cost (% of total)		
	Mean	Minimum	Maximum
Urea	22	16	31
MOP	16	10	27
DAP	30	15	40
Zinc sulfate	32	22	44

a Only the trials in which the RCM recommendation used all four sources (urea, MOP, DAP, and zinc sulfate) and no other sources are included.

Grain yield was consistently higher by 0.3–0.8 Mg ha^–1^ for RCM than FFP (Table 2), and grain yield was significantly higher by 0.2–0.4 Mg ha^–1^ for RCM than BFR in three of the four combinations of seasons and years. Some of the higher yield with RCM than FFP might be the result of overcoming zinc deficiency because zinc sulfate was added with RCM in all trials whereas zinc sulfate was added with FFP in only 5% of the trials. It cannot be determined from our study what proportion of the increase in yield from RCM compared to FFP was attributed to application of zinc. Higher yield with RCM than BFR was not associated with zinc because RCM and BFR received zinc sulfate at comparable rates.

At least some of the higher yield with RCM than FFP in kharif might be associated with higher use of fertilizer N with RCM in kharif (Table 2), resulting in significantly lower partial factor productivity of added N (PFP) for RCM than FFP in kharif 2014 (Table 2). Higher yield with RCM than BFR might be associated with higher use of fertilizer N with RCM for kharif and rabi, resulting in significantly lower PFP for RCM than BFR in three of the four combinations of seasons and years. The PFP with RCM nonetheless remained relatively high (46–54 kg grain per kg added N) across all seasons and years suggesting that fertilizer N was not overapplied with RCM (Dobermann, 2007).

**Fig. 2:**
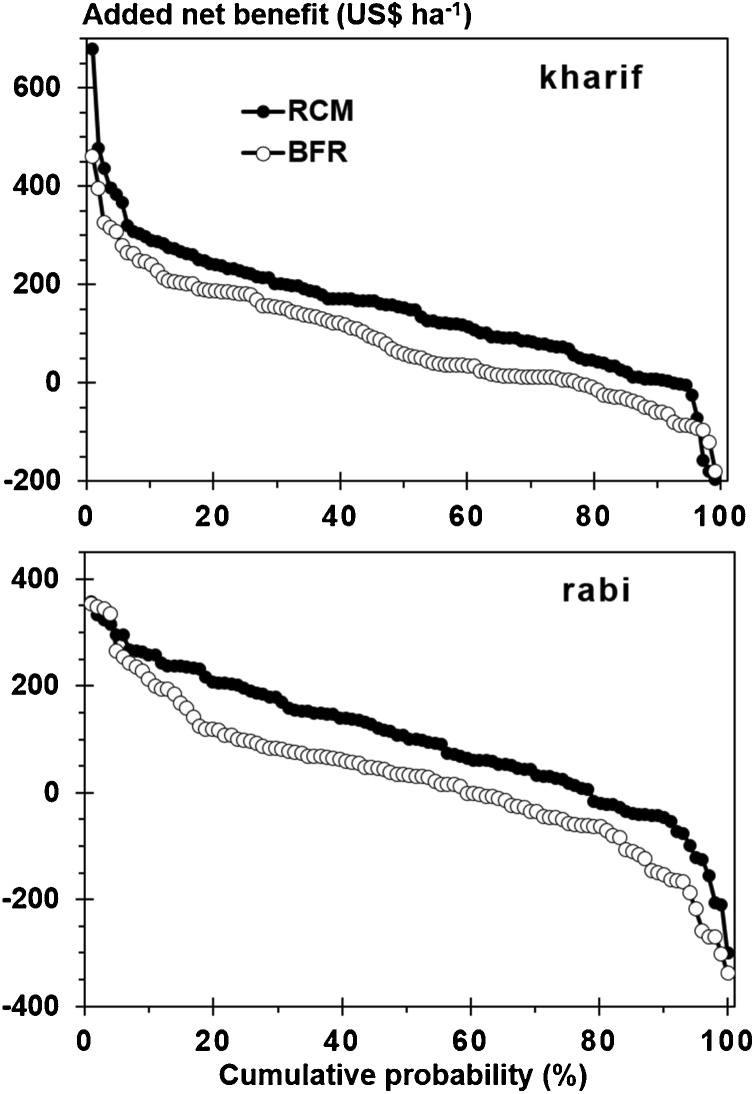
Cumulative probability of exceeding an added net benefit by switching from the farmers’ fertilizer practice (FFP) to either field-specific nutrient management provided by Rice Crop Manager (RCM) or blanket fertilizer recommendation (BFR) in two seasons (kharif and rabi) in Odisha, India.

Higher yields with RCM were usually not associated with higher TFC for RCM than for FFP or BFR and resulted in positive added net benefits with RCM. Added net benefit from use of RCM rather than FFP or BFR was consistently positive across seasons and years (Table 2). A switch from a farmers’ current fertilizer practice (FFP) to field-specific nutrient management (RCM) rather than uniform fertilizer application (BFR) had higher probability of financial gain and less risk of financial loss (Fig. 2). For example, with a switch from FFP to RCM the probability of obtaining ≥25 US$ ha^–1^ added net benefit was 84% in kharif and 74% in rabi. The probability was lower with a switch from FFP to BFR: 62% in kharif and 54% in rabi. The probability of financial loss (i.e., negative added net benefit) with a switch from FFP to RCM was 7% in kharif and 22% in rabi (Fig. 2). The probability of financial loss was higher with a switch from FFP to BFR: 23% in kharif and 41% in rabi.

**Fig. 3:**
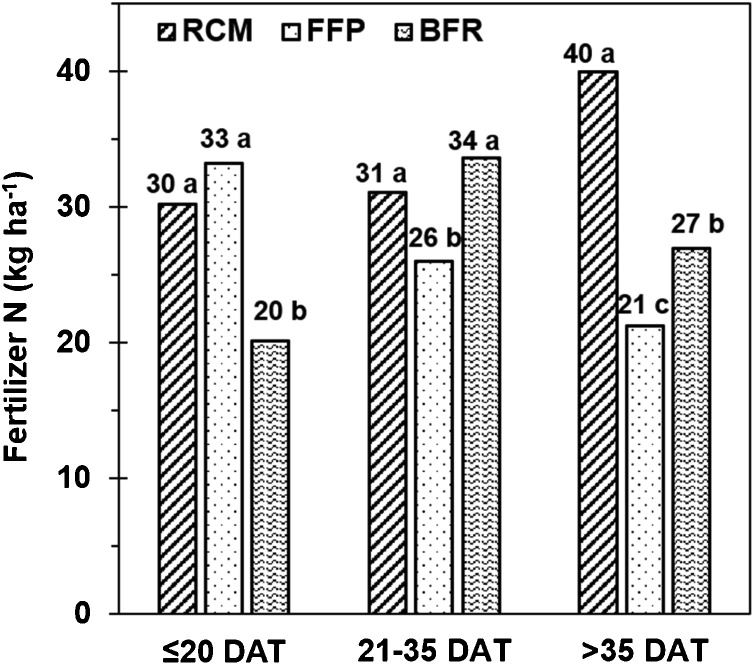
Amount of fertilizer N applied at three growth stages for rice, designated in days after transplanting (DAT), for field-specific nutrient management provided by Rice Crop Manager (RCM), farmers’ fertilizer practice (FFP), and blanket fertilizer recommendation (BFR) in Odisha, India. Means for a growth stage followed by the same letter are not different at P ≤ 0.05.

Differences in the application of fertilizer N during the growing season might have accounted for some of the differences in yield among RCM, FFP, and BFR. Fertilizer N was applied in three splits with RCM and BFR. With FFP the number of N applications varied from three in about 75% of the trials, two in about 20% of the trials, and one in <5% of the trials. FFP had the largest fraction of its total fertilizer N during early vegetative stage at ≤20 days after transplanting (DAT), and BFR had the largest fraction of its total fertilizer N during tillering at 21–35 DAT. RCM, on the other hand, had the largest fraction of its total fertilizer N at more than 35 days after transplanting (DAT), which included the time interval with panicle initiation. Total fertilizer N rate was higher for RCM than FFP and BFR. The actual amounts of fertilizer N applied at ≤20 DAT was therefore comparable for RCM and FFP (30 and 33 kg ha^–1^) but less for BFR (20 kg ha^–1^) (Fig. 3). The amounts of fertilizer N applied at 21–35 DAT was higher for RCM and BFR (31 and 34 kg ha^–1^) than FFP (26 kg ha^–1^), but much more N was applied with RCM (40 kg ha^–1^) than FFP and BFR (21 and 27 kg ha^–1^) after 35 DAT.

The mean yield achieved with RCM (4.8–5.5 Mg ha^–1^) matched or exceeded the RCM target yield (GY_T_) (4.2–5.1 Mg ha^–1^) (Table 2). This observation provided assurance the fertilizer N, P, and K rates with RCM were sufficient to achieve the target yield. The mean yield obtained by farmers (FFP) was within 0.2 Mg ha^–1^ of the historical yield reported by farmers during the RCM interview (GY_R_) in three of the four combinations of seasons and years (Table 2). This suggested a good match between a farmer’s estimate of previous yield and the yield achieved by a farmer in a subsequent season. The yield achieved with FFP in rabi 2013–2014, by contrast, was 1.5 Mg ha^–1^ higher than GY_R_ (Table 2). Significantly higher yield with FFP for rabi in 2013–2014 (5.2 Mg ha^–1^) than 2014–2015 (4.4 Mg ha^–1^) suggested more favorable crop-growing conditions in 2013–2014 than typical for rabi might have contributed to the higher yield with FFP than GY_R_.

Relatively low GY_R_ in rabi 2013–2014 (3.7 Mg ha^–1^) resulted in the setting of a relatively low RCM target yield (4.2 Mg ha^–1^), which contributed to the calculation of relatively lower fertilizer K rates with RCM (19 kg K ha^–1^) in that season (Table 2). The rates of fertilizer, including K, with RCM were nonetheless sufficient to achieve a yield 1.2 Mg ha^–1^ higher than targeted with RCM.

### 3.3 Evaluation across agro-climatic zones (ACZ)

The performance of an RCM recommendation relative to FFP and BFR was next examined across six ACZ in two seasons (Table 4). Grain yield in kharif was consistently higher for RCM than FFP (0.5–1.0 Mg ha^–1^) across all ACZ, except the five trials in 2014 in the north western plateau zone. Added net benefit from use of RCM rather than FFP was high (97–182 US$ ha^–1^) when yield was higher with RCM than FFP, but added net benefit was relatively lower (26 US$ ha^–1^) for the north western plateau zone in 2014 when yield was not higher with RCM.

**Table 4:** Measured rice grain yield and added net benefit (ANB) for field-specific nutrient management provided by Rice Crop Manager (RCM), farmers’ fertilizer practice (FFP), and blanket fertilizer recommendation (BFR) for two seasons (kharif and rabi) across six agro-climatic zones (ACZ) in Odisha, India.

Parameter	Season	Year	Treatment or contrast	East and south eastern coastal plain	Mid central table land	North central plateau	North eastern coastal plain	North western plateau	Western central table land
Trials (n)	Kharif	2014		25	5	24	25	5	0
		2015		6	5	5	0	3	5
	Rabi	2013–2014		17	0	10	12	0	0
		2014–2015		18	0	19	17	3	5
Grain yield (Mg ha^–1^)	Kharif	2014	RCM	6.1	5.9	4.7	6.0	4.2	
			FFP	5.2	5.1	3.7	5.4	4.0	
			BFR	6.0	5.1	4.4	5.5	4.1	
			RCM–FFP^a^	0.9^***^	0.8^**^	1.0^***^	0.6^***^	0.2 ns	
			RCM–BFR^b^	0.1 ns	0.8^**^	0.3 ns	0.5^***^	0.0 ns	
			BFR–FFP^c^	0.8^***^	0.0 ns	0.7^***^	0.1 ns	0.2 ns	
		2015	RCM	4.2	5.2	5.3		4.6	5.0
			FFP	3.5	4.5	4.8		3.9	4.5
			BFR	3.9	4.6	5.2		4.3	4.9
			RCM–FFP	0.7^***^	0.7^***^	0.5^**^		0.7^**^	0.5^**^
			RCM–BFR	0.3 ns	0.6^***^	0.0 ns		0.3 ns	0.1 ns
			BFR–FFP	0.4^*^	0.1 ns	0.5^**^		0.4 ns	0.3 ns
	Rabi	2013–2014	RCM	6.2		4.6	5.0		
			FFP	5.9		4.6	4.6		
			BFR	5.4		4.9	4.5		
			RCM–FFP	0.3 ns		0.0 ns	0.4 ns		
			RCM–BFR	0.8^***^		–0.3 ns	0.5^*^		
			BFR–FFP	–0.5^*^		0.3 ns	–0.1 ns		
		2014–2015	RCM	5.8		4.3	4.6	4.3	5.7
			FFP	5.0		4.0	4.1	3.8	5.5
			BFR	5.4		4.4	4.3	4.0	5.1
			RCM–FFP	0.8^***^		0.3^*^	0.6^***^	0.5 ns	0.2 ns
			RCM–BFR	0.4^***^		–0.2 ns	0.3^*^	0.3 ns	0.7^**^
			BFR–FFP	0.4^**^		0.5^***^	0.3^*^	0.2 ns	–0.4 ns
ANB (US$ ha^–1^)	Kharif	2014	RCM–FFP	177	132	174	128	26	
			RCM–BFR	31	172	51	101	18	
			Diff^d^	146^***^	–40 ns	123^***^	26 ns	8 ns	
		2015	RCM–FFP	126	99	97		147	182
			RCM–BFR	50	121	17		39	36
			Diff	76^**^	–22 ns	80^**^		108^**^	146^***^
	Rabi	2013–2014	RCM–FFP	76		–4	143		
			RCM–BFR	172		–37	132		
			Diff	–96^*^		33 ns	11 ns		
		2014–2015	RCM–FFP	177		34	139	101	60
			RCM–BFR	92		–33	69	69	138
			Diff	85^***^		68^**^	70^**^	32 ns	–78 ns

ns = not significant (P > 0.05). ^*^, ^**^, and ^***^ indicate significance at the 0.05, 0.01, and 0.001 probability levels, respectively.

a RCM–FFP = Difference between RCM and FFP.

b RCM–BFR = Difference between RCM and BFR.

c BFR–FFP = Difference between BFR and FFP.

d Diff = Difference between RCM–FFP and RCM–BFR.

RCM was less effective relative to FFP in rabi. Grain yield was not higher for RCM than FFP in the three ACZ with trials in 2013–2014, and grain yield was higher for RCM than FFP (0.3–0.8 Mg ha^–1^) in only three of the five ACZ with trials in 2014–2015 (Table 4). Added net benefit from use of RCM rather than FFP was low (34 US$ ha^–1^) or negative (–4 US$ ha^–1^) in the north central plateau zone but higher (60–177 US$ ha^–1^) for all other ACZ.

Higher yield with RCM than FFP was not associated with greater effectiveness in overcoming P or K deficiency because fertilizer P and K rates were never higher with RCM than FFP across all ACZ in both kharif and rabi (Table 5). Fertilizer N rate was higher with RCM than FFP across most ACZ in kharif, but fertilizer N rate was never higher with RCM than FFP within ACZ in rabi (Table 5). Higher yield with RCM than FFP in kharif might be associated with higher rates of N, improved timing of fertilizer N application during the growing season, and application of zinc with RCM. Higher yield with RCM than FFP in rabi, on the other hand, would not be associated with N rate, but it might be associated with improved split application of fertilizer N during the growing season and application of zinc with RCM.

**Table 5:** Rates of N, P, and K fertilizer for field-specific nutrient management provided by Rice Crop Manager (RCM), farmers’ fertilizer practice (FFP), and blanket fertilizer recommendation (BFR) for two seasons across six agro-climatic zones (ACZ) in Odisha, India.

Nutrient	Season	Year	Treatment or contrast	Nutrient rate (kg ha^–1^)					
				East and south eastern coastal plain	Mid central table land	North central plateau	North eastern coastal plain	North western plateau	Western central table land
N^a^	Kharif	2014	RCM	106	109	97	107	93	
			FFP	67	68	64	79	68	
			RCM–FFP^b^	38^***^	41^***^	35^***^	28^***^	25^*^	
		2015	RCM	101	119	104		95	109
			FFP	72	71	78		79	107
			RCM–FFP	28^***^	48^***^	26^**^		17 ns	2 ns
	Rabi	2013–2014	RCM	109		83	107		
			FFP	102		75	122		
			RCM–FFP	7 ns		8 ns	–15 ns		
		2014–2015	RCM	101		87	101	109	118
			FFP	104		74	97	94	127
			RCM–FFP	–3 ns		13 ns	5 ns	15 ns	–9 ns
P^a^	Kharif	2014	RCM	11	14	18	13	11	
			FFP	16	12	18	26	20	
			RCM–FFP	–5^**^	3 ns	0 ns	–13^***^	–9^*^	
		2015	RCM	12	13	12		12	12
			FFP	16	11	20		22	25
			RCM–FFP	–3^*^	2 ns	–7^***^		–11^***^	–13^***^
	Rabi	2013–2014	RCM	12		9	11		
			FFP	23		23	38		
			RCM–FFP	–11^***^		–14^***^	–27^***^		
		2014–2015	RCM	12		16	13	14	13
			FFP	26		18	29	25	25
			RCM–FFP	–14^***^		–2 ns	–16^***^	–11^*^	–11^**^
K^a^	Kharif	2014	RCM	27	35	27	30	25	
			FFP	44	35	33	48	27	
			RCM–FFP	–13^**^	0 ns	–6 ns	–18^***^	–2 ns	
		2015	RCM	47	33	29		27	30
			FFP	46	35	39		45	68
			RCM–FFP	1 ns	–2 ns	–10 ns		–18^*^	–37^***^
	Rabi	2013–2014	RCM	21		15	20		
			FFP	53		28	53		
			RCM–FFP	–32^***^		–13^*^	–34^***^		
		2014–2015	RCM	27		24	28	29	31
			FFP	57		23	48	37	62
			RCM–FFP	–30^***^		1 ns	–21^***^	–8 ns	–30^**^

ns = not significant (P > 0.05). ^*^, ^**^, and ^***^ indicate significance at the 0.05, 0.01, and 0.001 probability levels, respectively.

a Fertilizer applied with BFR: N = 80 kg ha^–1^, P =17 kg ha^–1^, and K =33 kg ha^–1^.

b RCM–FFP = Difference between RCM and FFP.

RCM tended to be more effective in increasing yield relative to BFR in rabi than kharif. Grain yield in kharif was higher for RCM than BFR (0.5–0.8 Mg ha^–1^) in only the mid central table land and north eastern coastal plain zones (Table 4), and added net benefit from use of RCM when compared to BFR was high (101–172 US$ ha^–1^) in these two ACZ. Added net benefit with RCM relative to BFR was 17–51 US$ ha^–1^ in other ACZ in kharif. In rabi, yield was higher for RCM than BFR (0.3–0.8 Mg ha^–1^) in all ACZ except north central plateau and north western plateau zones. Added net benefit from use of RCM relative to BFR was negative (–33 and –37 US$ ha^–1^) in the north central plateau zone but positive 69–172 US$ ha^–1^) in other ACZ.

Higher yield with RCM than BFR was not due to a higher P rate with RCM because mean fertilizer P rates across ACZ in kharif and rabi were not higher with RCM (9–18 kg P ha^–1^) (Table 5) than the uniform application of 17 kg P ha^–1^ with BFR. Higher yield with RCM than BFR was also not due to a higher K rate with RCM. Mean fertilizer K rate was distinctly higher with RCM than BFR in only the east and south eastern coastal plain in kharif 2015 (Table 5), but yield was not significantly higher in this ACZ and year with RCM (4.2 Mg ha^–1^) than BFR (3.9 Mg ha^–1^) (Table 4). Higher yield with RCM than BFR was also not associated with zinc because RCM and BFR received comparable uniform applications of zinc sulfate.

Mean fertilizer N rate for ACZ in kharif was higher with RCM (93–119 kg ha^–1^) (Table 5) than the uniform application on 80 kg N ha^–1^ with BFR. Higher yield with RCM than BFR in kharif in mid central table land and north eastern coastal plain zones (Table 4) might be associated with higher rate of N with RCM. In rabi, the mean fertilizer N rate for ACZ other than the north central plateau was higher with RCM (101–118 kg ha^–1^) (Table 5) than the uniform application on 80 kg N ha^–1^ with BFR. Higher yield with RCM than BFR in rabi for east and eastern coastal plain, north eastern coastal plain, and western central table land zones (Table 4) was likely associated with higher rate of N applied with RCM.

**Fig. 4:**
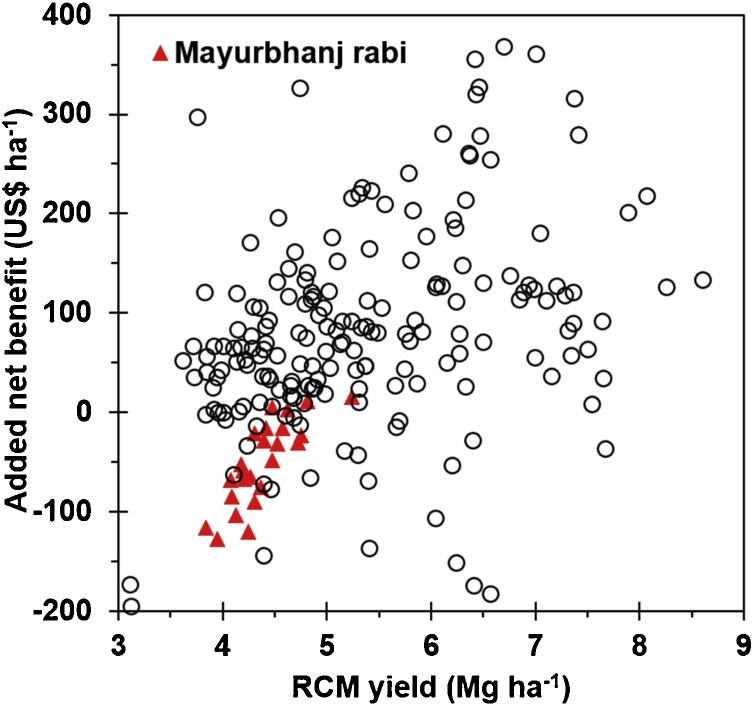
Relationship between rice grain yield with a Rice Crop Manager (RCM) recommendation and added net benefit for field-specific nutrient management provided by RCM relative to blanket fertilizer recommendation (BFR) in Odisha, India.

An RCM recommendation consistently failed to increase yield compared to BFR in kharif and rabi in the north central plateau zone, which had 29 trials each in kharif and rabi (Table 4). It was also the only zone with negative added net benefit with use of RCM (Table 4). Higher fertilizer N rate in kharif in this ACZ with RCM (97 and 104 kg N ha^–1^) (Table 5) than BFR (80 kg N ha^–1^) did not significantly increase yield with RCM, suggesting factors other than fertilizer N limited yield for RCM. The performance of RCM relative to BFR was especially poor in rabi in Mayurbhanj District, where 24 of the 29 rabi trials were conducted across the two years (Fig. 4). In Mayurbhanj District in rabi, 20 of the 24 trials had financial loss (negative added net benefit) with use of RCM rather than BFR (Fig. 4). These 20 trials represented 43% of the total number of field trials with negative added net benefit from use of RCM rather than BFR. Insufficient application of fertilizer P or K might be a factor limiting yield with RCM relative to BFR in Mayurbhanj District in rabi and perhaps other trials with negative added net benefit from use of RCM.

### 3.4 Evaluation across soils, water regime, and rice varieties

We examined whether the performance of an RCM recommendation relative to FFP and BFR was affected by soil clay content. Each of the field trials was placed into one of three categories for soil clay content based on texture. The low clay category included soils with sand, loamy sand, sandy loam, loam, and silty loam texture; the medium clay category included soils with clay loam, sandy clay loam, and silty clay loam texture; and high clay category included sandy clay, silty clay, and clay texture. The number of trials in each category was: low clay = 79, medium clay = 65, and high clay = 65. An ANOVA was conducted for grain yield and partial factor productivity of added N (PFP) across the three soil clay categories (C), three fertilizer treatments (T), and four cropping periods (CP; kharif 2014, kharif 2015, rabi 2013–2014, and 2014–2015) (Table 6).

**Table 6:** Analysis of variance for effect of cropping period, soil clay content, and fertilizer treatment on partial factor productivity of added N (PFP) and measured rice grain yield in Odisha, India.

SOV^a^	df^a^	Significance	
		PFP	Grain yield
Cropping period (CP)^b^	3	^***^	^***^
Soil clay (C)^c^	2	ns	ns
Treatment (T)^d^	2	^***^	^***^
CP × C	6	ns	^*^
CP × T	6	^*^	^***^
C × T	4	ns	ns
CP × C × T	12	ns	ns

ns = not significant (P > 0.05). *, **, and *** indicate significance at the 0.05, 0.01, and 0.001 probability levels, respectively.

a SOV = source of variation, df = degrees of freedom.

b Cropping periods are kharif 2014, kharif 2015, rabi 2013–2014, and 2014–2015.

c Soil clay categories are: low clay = sand, loamy sand, sandy loam, loam, and silty loam texture; medium clay = clay loam, sandy clay loam, and silty clay loam texture; and high clay = sandy clay, silty clay, and clay texture.

d Treatments are field-specific nutrient management provided by Rice Crop Manager (RCM), farmers’ fertilizer practice (FFP), and blanket fertilizer recommendation (BFR).

Clay category had no effect on grain yield and PFP; and differences among RCM, FFP, and BFR in grain yield and PFP were not affected by clay category as indicated by no significant C × T and CP × C × T interactions (Table 6). Grain yield was significantly higher for RCM than FFP in all four cropping periods regardless of soil clay content. Grain yield was significantly higher for RCM than BFR in only kharif 2014 and rabi 2013–2014 regardless of soil clay content (data not shown).

We further examined whether the performance of an RCM recommendation relative to FFP and BFR was affected by water regime (irrigated or rainfed). This analysis was limited to kharif for 2014 and 2015 because all trials in rabi were irrigated. The number of trials was: irrigated = 48 and rainfed = 60. An ANOVA for the two water regimes (W) across the two years (Y) and fertilizer treatments (T) revealed a significant Y × W × T interaction for grain yield across the three fertilizer treatments (RCM, FFP, and BFR) and a significant W × T interaction for fertilizer N and P rates across the two fertilizer treatments (RCM and FFP) with variable rates of fertilizer (Table 7).

**Table 7:** Analysis of variance for effect of water regime and treatment in kharif on rates of N, P, and K fertilizer, partial factor productivity of added N (PFP), and measured rice grain yield in Odisha, India.

SOV^a^	df^a^	Significance			df	Significance	
		N rate	P rate	K rate		PFP	Grain yield
Year (Y)^b^	1	^*^	ns	ns	1	^***^	^*^
Water (W)^c^	1	ns	^*^	^*^	1	ns	ns
Treatment (T)^d^	1	^***^	^***^	^***^	2	^***^	^***^
Y × W	1	ns	ns	ns	1	ns	ns
Y × T	1	ns	ns	ns	2	ns	ns
W × T	1	^*^	^*^	ns	2	ns	ns
Y × W × T	1	ns	ns	ns	2	ns	^**^

ns = not significant (P > 0.05). ^*^, ^**^, and ^***^ indicate significance at the 0.05, 0.01, and 0.001 probability levels, respectively.

a SOV = source of variation, df = degrees of freedom.

b Years are 2014 and 2015.

c Water regimes are irrigated and rainfed.

d Treatments are field-specific nutrient management provided by Rice Crop Manager (RCM) and farmers’ fertilizer practice (FFP) for N, P, and K rates; and RCM, FFP, and blanket fertilizer recommendation (BFR) for PFP and grain yield.

RCM was equally effective across irrigated and rainfed trials in increasing yield relative to FFP. Grain yield was consistently higher with RCM than FFP (0.6–0.9 Mg ha^–1^) in both irrigated and rainfed conditions across the two years (Table 8). This resulted in relatively high added net benefits (95–172 US$ ha^–1^) with use of RCM rather than FFP across water regimes and years.

**Table 8:** Influence of water regime in kharif on rate of N and P fertilizer, rice grain yield, and added net benefit (ANB) for field-specific nutrient management provided by Rice Crop Manager (RCM), farmers’ fertilizer practice (FFP), and blanket fertilizer recommendation (BFR) in Odisha, India.

Parameter	Year	Treatment or contrast	Water regime	
			Irrigated	Rainfed
Trials (n)	2014		31	53
	2015		17	7
N rate (kg ha^–1^)^a^		RCM	104	107
		FFP	80	70
		RCM–FFP^b^	24^***^	37^***^
P rate (kg ha^–1^)^a^		RCM	13	13
		FFP	22	16
		RCM–FFP	–8^***^	–3 ns
Grain yield (Mg ha^–1^)	2014	RCM	5.6	5.5
		FFP	5.1	4.5
		BFR	5.2	5.3
		RCM–FFP	0.6^***^	0.9^***^
		RCM–BFR^c^	0.4^***^	0.2^**^
		BFR–FFP^d^	0.1 ns	0.7^***^
	2015	RCM	4.8	4.9
		FFP	4.2	4.3
		BFR	4.6	4.4
		RCM–FFP	0.6^***^	0.6^*^
		RCM–BFR	0.2 ns	0.5 ns
		BFR–FFP	0.4^**^	0.1 ns
ANB (US$ ha^–1^)	2014	RCM–FFP	112	172
		RCM–BFR	91	50
		Diff^e^	21 ns	122^***^
	2015	RCM–FFP	143	95
		RCM–BFR	39	90
		Diff	104^***^	5 ns

ns = not significant (P > 0.05). ^*^, ^**^, and ^***^ indicate significance at the 0.05, 0.01, and 0.001 probability levels, respectively.

a Fertilizer applied with BFR: N = 80 kg ha^–1^, P =17 kg ha^–1^, and K =33 kg ha^–1^.

b RCM–FFP = Difference between RCM and FFP.

c RCM–BFR = Difference between RCM and BFR.

d BFR–FFP = Difference between BFR and FFP.

e Diff = Difference between RCM–FFP and RCM–BFR.

Fertilizer N and P rates with RCM were unaffected by water regime (Table 8). This indicates RCM target yield was comparable for irrigated and rainfed trials because RCM fertilizer rates are directly related to target yield. Fertilizer P rates were lower with RCM than FFP for irrigated but not rainfed trials because fertilizer P with FFP tended to be lower for rainfed than irrigated trials (Table 8). Higher yield with RCM than FFP across water regimes might be associated with higher rates of N, improved split application of fertilizer N during the growing season, and application of zinc with RCM. It was not associated with P or K rates because they were not higher for RCM than FFP.

The performance of RCM relative to BFR was not consistent for the two years. Grain yield was higher with RCM than BFR for both irrigated and rainfed conditions in 2014 but not in 2015 when a much lower number of trials were conducted (Table 8). Added net benefits were positive for use of RCM rather than BFR (39–91 US$ ha^–1^), but added net benefits for RCM relative to BFR were never higher than added net benefits for RCM relative to FFP.

The performance of BFR relative to FFP across water regime was inconsistent. Grain yield was higher for BFR than FFP with rainfed trials but not irrigated trials in 2014 (Table 8). The trend was reversed in 2015 when grain yield was higher for BFR than FFP with irrigated trials but not rainfed trials.

We also examined whether the performance of an RCM recommendation relative to FFP and BFR was affected by the growth duration of rice variety. Each of the field trials was placed into a category based on growth duration of the rice variety from germination to maturity. The categories were: short defined as ≤ 120 days, medium defined as 121–140 days, and long defined as >140 days. Medium duration varieties are common in kharif and rabi, whereas long duration varieties are common in kharif but not rabi and short duration varieties are common in rabi but rare in kharif. The number of trials in kharif was: medium duration = 45 and long duration = 54. The number of trials in rabi was: short duration = 32 and medium duration = 41.

Separate ANOVA for kharif and rabi with two categories for growth duration (V) across two years (Y) and the three fertilizer treatments (T) revealed no effect of variety duration category on grain yield or partial factor productivity of added N (Table 9). Differences among RCM, FFP, and BFR in grain yield and partial factor productivity were not affected by variety duration category as indicated by no significant V × T and Y × V × T interactions (Table 9). Grain yield was significantly higher for RCM than FFP and for RCM than BFR in kharif and rabi regardless of variety duration category (data not shown).

**Table 9:** Analysis of variance for effect of duration of rice varieties and treatment in kharif and rabi on partial factor productivity of added N (PFP) and measured rice grain yield in Odisha, India.

SOV^a^	df^a^	Significance			
		Kharif		Rabi	
		PFP	Grain yield	PFP	Grain yield
Year (Y)^b^	1	^***^	^*^	^*^	^***^
Variety (V)^c^	1	ns	ns	ns	ns
Treatment (T)^d^	2	^***^	^***^	^**^	^***^
Y × V	1	^*^	^*^	ns	ns
Y × T	2	^*^	ns	ns	^***^
V × T	2	ns	ns	ns	ns
Y × V × T	2	ns	ns	ns	ns

ns = not significant (P > 0.05). ^*^, ^**^, and ^***^ indicate significance at the 0.05, 0.01, and 0.001 probability levels, respectively.

a SOV = source of variation, df = degrees of freedom.

b Years for kharif are 2014 and 2015, and years for rabi are 2013–2014 and 2014–2015.

c Varieties are medium and long growth duration in kharif and short and medium growth duration in rabi.

d Treatments are field-specific nutrient management provided by Rice Crop Manager (RCM), farmers’ fertilizer practice (FFP), and blanket fertilizer recommendation (BFR).

## 4 Discussion

### 4.1 Performance of RCM

Higher yield with an RCM recommendation than BFR and positive added net benefit with RCM relative to BFR (Table 2) were attributed to improved N management because zinc fertilizer rates were comparable for RCM and BFR and P and K rates were not higher for RCM than BFR. Much of the improvement in N management with RCM can be associated with the ability of RCM to adjust fertilizer N rate upward when N limited the achievement of higher yield. BFR used a constant fertilizer N rate of 80 kg ha^–1^ across all trials in both kharif and rabi. RCM estimated 80 kg N ha^–1^ to be sufficient for a target yield of 4 Mg ha^–1^, which corresponded to a partial factor productivity of fertilizer N of 50 kg grain kg^–1^ N. RCM adjusted fertilizer N rates upward or downward based on a field-specific target yield, resulting in application between 54–143 kg N ha^–1^ (Supplement Table 4) and the targeting of a partial factor productivity approximating 50 kg grain kg^–1^ N across the range of 3.0–6.5 Mg ha^–1^.

The partial factor productivity of fertilizer N for RCM in our study (46–54 kg grain kg^–1^ N) (Table 2) generally achieved a targeted 50 kg grain kg^–1^ N. It was comparable to the mean of 52 kg grain kg^–1^ N reported for SSNM across 179 on-farm trials with irrigated rice in six Asian countries (Dobermann et al., 2002) and higher than the 30–44 kg grain kg^–1^ N reported with NMR in Tamil Nadu (Sharma et al., 2019). The partial factor productivity in our study suggested fertilizer N rates with RCM were not excessive, whereas the relatively higher partial factor productivity with BFR (56–65 kg grain kg^–1^ N) might represent a risk that fertilizer N with BFR was at least occasionally insufficient to eliminate N as the main limiting factor.

Higher yield with RCM than BFR as well as FFP could be partially associated with improved split application of fertilizer N to better match the demand of the rice crop for supplemental N. The farmers in our study, as reported earlier for other rice farmers across Asia (Dobermann et al., 2002; Peng et al., 2006), applied a large proportion of the total fertilizer N at the early vegetative stage when the rice crop had limited capacity to utilize added N. RCM in contrast distributed fertilizer N more uniformly into the late vegetative stage and early reproductive stage (Fig. 3) to ensure adequate N at panicle initiation for increasing spikelets per panicle. Banayo et al. (2018a) reported a larger application of N at panicle initiation with RCM than FFP was a critical factor for higher rice yield with RCM. More fertilizer N with RCM and BFR than FFP at 21–35 DAT and >35 DAT (Fig. 3) suggested that improved split application of fertilizer N accounted for some of the higher yield with RCM and BFR than FFP. More N at >35 DAT with RCM than BFR might have accounted for some of the higher yield with RCM than BFR, but we cannot in our study separate the relative contributions of higher N rates and improved split application of fertilizer N to higher yield with RCM than BFR.

RCM for Odisha had the capability, as described by Sharma et al. (2019) for NMR in Tamil Nadu, to adjust N applications to match with tillering and panicle initiation stages across rice varieties with a wide range in growth durations and ages of transplanted seedlings. Consistently higher yield with RCM than BFR and FFP across rice varieties with different growth duration (Table 9) provided confidence in the ability of RCM to effectively adjust the split application and timing of fertilizer N to match critical growth stages of rice.

Yields for irrigated and rainfed rice were comparable with RCM (Table 8) suggesting that water deficit or excess at rainfed sites had negligible effect on yield. Higher yields with RCM than BFR and FFP across irrigated and rainfed rice (Table 7, Table 8) provided encouragement for use of RCM across irrigated and rainfed rice with limited or no water stress. Our study, however, has not thoroughly assessed the capability of RCM to adjust target yield and fertilizer use for rainfed rice with risk of yield reduction due to water deficit stress.

A noteworthy feature of rice production in Odisha was the wide range in rice yields. Farmers in our study reported previous yields (GY_R_) between 1.7–6.3 Mg ha^–1^, and they achieved yields between 2.8–7.9 Mg ha^–1^ with farmers’ practice (FFP) across two seasons for two years. BFR with a uniform fertilizer application failed in 18% of the trials to achieve >4.0 Mg ha^–1^ suggesting that factors other than nutrients such as farmers’ farming practices, weather, water supply, and yield potential of the rice variety sometimes limited yield. On the other hand, yield as high as 8.0 Mg ha^–1^ was achieved with BFR indicating that uniform nutrient management could sometimes achieve high yield when other factors including native soil fertility were not limiting.

The ability of RCM, unlike BFR, to adjust fertilizer use and costs with a target yield based on field-specific rice-farming practices and conditions likely contributed to the reduced risk of financial loss with a switch from FFP to RCM rather than BFR (Fig. 2). Sharma et al. (2019) similarly observed reduced risk of financial loss with a switch from FFP to field-specific nutrient management rather than to a uniform fertilizer application with irrigated rice in Tamil Nadu. Reduced risk of financial loss might be an attribute of RCM that can help convince risk-averse farmers to change existing fertilizer practices.

Zinc can be an important limiting nutrient for rice because the submergence of non-calcareous soil in rice production tends to reduce the availability of zinc (Dobermann and Fairhurst, 2000). Chemical extractants have been used to determine available soil zinc, but their reliability in identifying zinc deficiency has been questioned because factors unrelated to the chemical extraction, such as soil drainage and rice variety also influence the occurrence of zinc deficiency (Neue et al., 1998; Impa and Johnson-Beebout, 2012). The SSNM developed for rice by Dobermann et al. (2004b) was limited to N, P, and K; and RCM for Odisha did not include the capability to adjust zinc use across rice fields. The RCM recommendations in our study used a uniform application of zinc sulfate at all locations in each season and year. This eliminated zinc as a factor affecting the difference in performance between RCM and BFR, but zinc sulfate represented a large proportion (32%) of the total fertilizer cost with RCM (Table 3). The higher cost for zinc sulfate than urea with RCM (Table 3) highlighted the need to reduce cost for zinc fertilization by incorporating into RCM a capability to adjust use of zinc fertilizer for location-specific factors such as water regime, rice variety, and growing season (kharif and rabi) as well as soil to ensure use of zinc fertilizer where zinc deficiency is likely to occur.

### 4.2 Lessons for improvement of RCM

Nutrient Manager for Rice (NMR), which preceded RCM, used algorithms adapted from principles of SSNM together with information on a farmers’ rice-farming practices to calculate rates and application times for fertilizer N, P, and K (Sharma et al., 2019). RCM expanded NMR by providing a web-based platform for integrating the calculation of nutrient management with an automated selection of improved crop management practices for a farmer. Our study was limited to an evaluation of only the nutrient management component of RCM adapted for rice-farming practices, rice varieties, and rice-growing conditions in Odisha, India.

The adaptation of the SSNM-based nutrient management component of RCM for a rice-growing region, such as Odisha, includes the calibration of the calculation of fertilizer rates using results from NOPT trials (Dobermann et al., 2003; Buresh et al., 2019). When the development of RCM for Odisha started in 2013, results from NOPT trials conducted in Odisha were not available. Experiences with SSNM and results for NOPT trials from outside Odisha were consequently used to develop the initial version of calculations for fertilizer N, P, and K rates, which was evaluated in this study. Our study can therefore provide valuable insights and lessons into fast tracking the development and evaluation of an initial version of RCM before results from NOPT trials are available for the rice-growing region or domain dealt with by RCM.

Higher yield with RCM than BFR, attributed to improved N management, suggested the split application of fertilizer N to match critical growth stages and the calculation of N rates using an SSNM-based, yield-gain approach targeting a realistically achievable PFP could be an appropriate starting point with RCM before additional data are available for calibrating the calculation of N rate. The use of information on estimated duration of a rice variety and expected age of transplanted rice seedlings to match N application with critical growth stages, as used in RCM for Odisha and described in more detail by Sharma et al. (2019), provides a guideline that can be used elsewhere.

The setting of a target yield, which increases net income and can be achieved with little risk of failure by farmers, is vital to the effectiveness of field-specific N management because calculated N rate is directly related to target yield. RCM was able to match or exceed target yield in our study (Table 2) suggesting that our approach of setting target yield slightly higher than historical yield reported by farmers while adjusting target yield downward for late transplanting can be an effective starting point for an initial version of RCM. Future research and subsequent upgrades of RCM can fine tune the setting of target yield through use of factors such as socioeconomic characteristics of a farmer, management skills and practices of the farmer, historical climate, forecasted weather, and yield potential of the rice variety.

RCM for Odisha used a nutrient input-output balance to calculate P rate because research by Sharma et al. (2019) indicated this could be effective for field-specific nutrient management. With this approach a field-specific P rate matches the estimated output of P with harvested grain and removed crop residue. It avoids risk of soil P depletion while often reducing P rates for existing recommendations and for farmers’ practice as observed in our study (Table 2). It can risk insufficient application of P on P-fixing soils, but such soils are not common in lowland rice production.

Fertilizer K rates can be determined using either a nutrient input-output balance or a yield-gain approach (Buresh et al., 2010, 2019). Differences exist among decision tools in the approach used. RCM for Odisha used a yield-gain approach, but NMR for Tamil Nadu used a nutrient-balance approach (Sharma et al., 2019) and RCM for the Philippines used a combined yield-gain + nutrient-balance approach (Buresh et al., 2019). When crop residues are removed, a yield-gain approach often recommends lower rates of fertilizer K than a nutrient-balance approach, thereby favoring short-term financial gain at the risk of long-term depletion of soil K (Buresh et al., 2010).

Low rice yield with RCM relative to BFR in the north central plateau zone (Table 4), and particularly in Mayurbhanj District in rabi (Fig. 4), suggested that RCM failed to overcome P and/or K deficiencies. Research and NOPT trial data are needed to appropriately adjust fertilizer P and/or K rates in this rice-growing area. One option is a domain-specific adjustment in which fertilizer rates across all fields within a geographical area and season, as determined by the domain, are uniformly adjusted for a target yield. Another option is a field-specific adjustment in which fertilizer rates are adjusted only for fields with specific characteristics shown to be associated with the need for additional nutrient and identifiable by farmers during the interview with RCM. The absence of an effect of soil clay content on effectiveness of RCM relative to BFR and FFP (Table 6) suggested soil texture was probably not an effective indictor of soils requiring additional P or K than calculated by RCM.

## 5 Conclusions

Field-specific nutrient management as calculated by RCM for Odisha uses principles and algorithms adapted from SSNM to provide rice farmers with an alternative to the uniform application of nutrients. Field-specific nutrient management was developed on the premise that spatially variable factors in addition to soils—such as rice variety, water regime, crop sequence, crop management practices, date of rice establishment, age of rice seedlings at transplanting, and socioeconomic status of farmers—can influence optimal fertilization. The 209 farmers in our study reported previous yields between 1.7–6.3 Mg ha^–1^, and they achieved yields between 2.8–7.9 Mg ha^–1^ with their farming practices.

High variability in yields across small landholdings suggested that Odisha was well suited for field-specific nutrient management, which calculates fertilizer rates based on a target yield adjusted for historical yield reported by farmers. Higher yields, positive net benefits, and less risk of financial loss with an RCM recommendation than BFR confirm the merits of a field-specific nutrient management. Our findings in Odisha could be relevant to other rice-growing areas with high spatial and temporal variability in factors affecting optimal fertilization.
