# Supplementary material for: Field-specific nutrient management using Rice Crop Manager decision support tool in Odisha, India
Source: Field Crops Res. 2019 Sep 1;241:107578. doi: 10.1016/j.fcr.2019.107578 (PMC6737991; doi:10.1016/j.fcr.2019.107578)
Supplement: Supplementary file 1 [file mmc1.docx]

**Supplementary tables**

**Article title:**

Field-specific nutrient management using Rice Crop Manager decision support tool in Odisha, India

**Journal name:**

Field Crops Research

**Author names:**

Sheetal Sharma^a^, R.K. Rout^d^, C.M. Khanda^d^, Rahul Tripathi^e^, Mohammad Shahid^e^, Amarash Nayak^e^, Swetapadma Satpathy^a^, Narayan Chandra Banik^a^, Wasim Iftikar^c^, Nabakishore Parida^c^, Vivek Kumar^a^, Amit Mishra^a^, Rowena L. Castillo^b^, Theresa Velasco^b^, Roland J. Buresh^b^

**Author affiliations:**

^a^ International Rice Research Institute, India

^b^ International Rice Research Institute, PO Box 7777, Metro Manila, Philippines

^c^ CIMMYT, India

^d^ Odisha University of Agriculture and Technology (OUAT), Bhubaneswar, India

^e^ National Rice Research Institute (NRRI), Cuttack, Odisha, India

**Email address of corresponding author:**

Sheetal Sharma^:^ [Sheetal.sharma@irri.org](mailto:Sheetal.sharma@irri.org)

**Content**

Supplement Table 1. Numbers of field trials by district, season (kharif and rabi), and year across six agro-climatic zones (ACZ) in Odisha, India.

Supplement Table 2. Rice varieties grown each season (kharif and rabi) in field trials across Odisha, India.

Supplement Table 3. Fertilizer sources applied with Rice Crop Manager (RCM), farmers’ fertilizer practice (FFP), and blanket fertilizer recommendation (BFR) in Odisha, India.

Supplement Table 4. Range in N, P, and K fertilizer rates for field-specific nutrient management provided by Rice Crop Manager (RCM) and farmers’ fertilizer practice (FFP) for two seasons (kharif and rabi) in Odisha, India.

Supplement Table 1. Numbers of field trials by district, season (kharif and rabi), and year across six agro-climatic zones (ACZ) in Odisha, India.

| ACZ | District | Number of field trials | | | | | | | |
| --- | --- | --- | --- | --- | --- | --- | --- | --- | --- |
|  |  | Kharif | | |  | | Rabi | | |
|  |  | 2014 | 2015 |  | | 2013–2014 | | 2014–2015 |  |
| East and south eastern coastal plain | Kendrapara | 6 | 6 |  | | – | | 4 |  |
|  | Puri | 19 | – |  | | 17 | | 14 |  |
| Mid central table land | Dhenkanal | 5 | 5 |  | | – | | – |  |
| North central plateau | Keonjhar | 5 | 5 |  | | – | | 5 |  |
|  | Mayurbhanj | 19 | – |  | | 10 | | 14 |  |
| North eastern coastal plain | Balasore | 4 | – |  | | – | | 5 |  |
|  | Bhadrak | 17 | – |  | | 12 | | 12 |  |
|  | Jajpur | 4 | – |  | | – | | – |  |
| North western plateau | Sundargarh | 5 | 3 |  | | – | | 3 |  |
| Western central table land | Sambalpur | – | 5 |  | | – | | 5 |  |

Supplement Table 2. Rice varieties grown each season (kharif and rabi) in field trials across Odisha, India.

| Rice variety | Approximate duration (d) | Number of field trials | |
| --- | --- | --- | --- |
|  |  | Kharif | Rabi |
| Annapoorna | 101–110 | 1 | – |
| CR 1009 | 141–150 | 2 | – |
| CR Dhan 403 | 131–140 | 1 | – |
| Jagabandhu | 141–150 | 2 | – |
| Kalashree | 151–160 | 3 | – |
| Khandagiri | 91–100 | – | 10 |
| Konark | 121–130 | – | 3 |
| Lalat | 121–130 | 14 | 49 |
| MTU 1001 | 125–130 | 3 | 2 |
| MTU 1010 | 120–125 | 3 | 7 |
| Naveen | 121–130 | 8 | 6 |
| Parijat | 91–100 | – | 9 |
| Pioneer 27P31 (hybrid) | 121–130 | 1 | – |
| Pooja | 141–150 | 14 | – |
| Pratikshya | 141–150 | 5 | – |
| Rajalaxmi (hybrid) | 135–140 | 1 | – |
| Ranidhan | 141–150 | 5 | – |
| Sahbhagi dhan | 105–110 | – | 9 |
| Sarala (photosensitive) | >160 | 2 | – |
| Shankar | 91–100 | – | 3 |
| Suruchi 5629 (hybrid) | 101–110 | – | 2 |
| Swarna | 141–150 | 38 | – |
| Swarna–Sub1 | 141–150 | 4 | – |
| Upahar | 161–170 | 1 | – |

Supplement Table 3. Fertilizer sources applied with Rice Crop Manager (RCM), farmers’ fertilizer practice (FFP), and blanket fertilizer recommendation (BFR) in Odisha, India.

| Source^a^ | Trials using source (%)^b^ | | |  | Amount of source when used (kg ha^–1^)^c^ | | | | | | | | | |
| --- | --- | --- | --- | --- | --- | --- | --- | --- | --- | --- | --- | --- | --- | --- |
|  |  |  |  |  | Minimum | | |  | | Maximum | | | |  |
|  | RCM | FFP | BFR |  | RCM | FFP | BFR | |  | | RCM | FFP | BFR | |
| Urea | 100 | 99 | 100 |  | 97 | 32 | 112 | |  | | 283 | 423 | 188 | |
| MOP | 100 | 94 | 100 |  | 10 | 21 | 37 | |  | | 98 | 312 | 76 | |
| DAP | 85 | 82 | 86 |  | 31 | 25 | 77 | |  | | 107 | 318 | 99 | |
| SSP | 1.4 | 1.0 | 14 |  | 120 | 49 | 207 | |  | | 207 | 247 | 270 | |
| 28-28-0 | 13 | 15 | – |  | 78 | 62 | – | |  | | 125 | 333 | – | |
| 10-26-26 | 0.5 | 0.5 | – |  | 135 | 247 | – | |  | | 135 | 247 | – | |
| 20-20-0 | – | 1.0 | – |  | – | 63 | – | |  | | – | 124 | – | |

^a^ MOP = Muriate of potash or KCl (0-0-60), DAP = Diammonium phosphate (18-46-0), and SSP = Single superphosphate. The N-P-K content for compound fertilizers is expressed on a P_2_O_5_ and K_2_O basis.

^b^ Percentage of total field trials (n = 209) in which a source was applied.

^c^ Minimum and maximum refer to amount of a source applied when the source was used in a field trial.

Supplement Table 4. Range in N, P, and K fertilizer rates for field-specific nutrient management provided by Rice Crop Manager (RCM) and farmers’ fertilizer practice (FFP) for two seasons (kharif and rabi) in Odisha, India.

| Treatment | Season^a^ | Nutrient^b^ | Nutrient rate (kg ha^–1^) | | | | | |
| --- | --- | --- | --- | --- | --- | --- | --- | --- |
|  |  |  | Mean | Min^c^ | 25% quartile | Median | 75% quartile | Max^c^ |
| RCM | Kharif | N | 104 | 79 | 99 | 102 | 111 | 130 |
|  |  | P | 14 | 6 | 11 | 13 | 15 | 21 |
|  |  | K | 30 | 19 | 26 | 28 | 31 | 49 |
|  |  |  |  |  |  |  |  |  |
|  | Rabi | N | 100 | 54 | 80 | 102 | 113 | 143 |
|  |  | P | 13 | 7 | 11 | 13 | 14 | 18 |
|  |  | K | 24 | 12 | 21 | 23 | 28 | 32 |
|  |  |  |  |  |  |  |  |  |
| FFP | Kharif | N | 72 | 29 | 57 | 73 | 85 | 136 |
|  |  | P | 19 | 0 | 14 | 18 | 25 | 43 |
|  |  | K | 41 | 0 | 27 | 38 | 50 | 108 |
|  |  |  |  |  |  |  |  |  |
|  | Rabi | N | 97 | 14 | 78 | 93 | 119 | 252 |
|  |  | P | 26 | 5 | 20 | 25 | 27 | 64 |
|  |  | K | 45 | 0 | 31 | 46 | 62 | 156 |

^a^ Number for trials: kharif = 108, rabi = 101.

^b^ P and K are expressed on an elemental basis.

^c^ Min = minimum, Max = maximum.
